# Inside of the Linear Relation between Dependent and Independent Variables

**DOI:** 10.1155/2015/360752

**Published:** 2015-05-25

**Authors:** Lorentz Jäntschi, Lavinia L. Pruteanu, Alina C. Cozma, Sorana D. Bolboacă

**Affiliations:** ^1^Institute for Doctoral Studies, Technical University of Cluj-Napoca, Muncii Boulevard 103-105, 400641 Cluj-Napoca, Romania; ^2^Institute for Doctoral Studies, Babeş-Bolyai University, Kogălniceanu Street No. 1, 400084 Cluj-Napoca, Romania; ^3^Department of Chemistry, University of Oradea, Universităţii Street No. 1, 410087 Oradea, Romania; ^4^Department of Medical Informatics and Biostatistics, Iuliu Haţieganu University of Medicine and Pharmacy, Louis Pasteur Street No. 6, 400349 Cluj-Napoca, Romania

## Abstract

Simple and multiple linear regression analyses are statistical methods used to investigate the link between activity/property of active compounds and the structural chemical features. One assumption of the linear regression is that the errors follow a normal distribution. This paper introduced a new approach to solving the simple linear regression in which no assumptions about the distribution of the errors are made. The proposed approach maximizes the probability of observing the event according to the random error. The use of the proposed approach is illustrated in ten classes of compounds with different activities or properties. The proposed method proved reliable and was showed to fit properly the observed data compared to the convenient approach of normal distribution of the errors.

## 1. Introduction

The quantitative structure activity/property relationships (QSARs/QSPRs) are computational techniques that quantitatively relate chemical feature (such as descriptors) to a biological activity or property [1]. Linear regression is one of the earliest methods [2] used to link the activity/property with structural information and is frequently used due to the relative easy interpretation [3]. Sometimes, linear regression is misuse due to the application without investigation of its assumptions (such as linearity, independence of the errors, normality, homoscedasticity, and absence of multicollinearity [4]).

The error, “a measure of the estimated difference between the observed or calculated value of a quantity and its true value” [5], was first used in mathematics/statistics in 1726 in* Astronomiae Physicae & Geometricae Elementa* [6]. In the late 1800's, Adcock [7, 8] suggested that the errors must pass through the centroid of the data. The method proposed by Adcock, named orthogonal regression, explores the distance between a point and the line in a perpendicular direction to the line [7, 8]. Kummell [9] investigated other than perpendicular directions between the points and line. The regression slope (“*r*”) was described by Galton in 1894 based on an experiment of sweet pea seeds [10]. Two years later, Pearson generalized the errors in the variable and published a rigorous description of correlation and regression analysis [11] (Pearson recognized the contribution of Bravais [12] to mathematical formula of correlation). Due to the ability to produce best linear unbiased parameters [13], the coefficients in simple linear regression (SLR) models are estimated by minimizing the sum of squared deviations (least squares estimation, method introduced by Legendre in 1805 [14] and used/applied by Gauss in 1809 [15]). Furthermore, Fisher introduced the concept of maximum likelihood within linear models [16, 17].

The generic equation of simple linear regression (1) between observed dependent variable *Y* and observed independent variable *X* is:

(1) $$\begin{array}{c} Y\sim \hat{Y}=a\cdot X+b, \end{array}$$

where *a* and *b* are unknown constant values (estimators of statistics parameters of simple linear regression), $\hat{Y}$ is the value of the dependent variable estimated by the model, *Y* is the observed value of dependent variable, and *X* is the observed value of the predictor variable.

The array use to estimate the residuals is given by (*Y*_*i*_ − *a* · *X*_*i*_ − *b*)^*q*^ formula, where *i* is the *i*th observation in the sample (1 ≤ *i* ≤ *n*, when *n* = sample size) and *q* is an unknown coefficient. The unknown *q* coefficient is an estimator of the power of the errors on simple linear regression.

In the SLR-LS (simple linear regression least squares), residuals (*S*_*i*_ = *Y*_*i*_ − *aX*_*i*_ − *b*, where *S* = residual) follow the Gauss-Laplace distribution with *μ*, *σ*, and *q* being unknown statistical parameters:

(2) $$\begin{array}{c} \text{GL}\left(s;\mu ,\sigma ,q\right)=\frac{q}{2\sigma }\frac{\Gamma^{1/2}\left(3/q\right)}{\Gamma^{3/2}\left(1/q\right)}\exp\left(-\frac{{\left|\left(s-\mu \right)/\sigma \right|}^{q}}{{\left(\Gamma \left(1/q\right)/\Gamma \left(3/q\right)\right)}^{q/2}}\right), \end{array}$$

where *μ* is population mean, *σ* is population standard deviation, *q* is power of the errors, Γ is gamma function, and *s* is sample standard deviation.

Gauss-Laplace distribution is symmetrical and has three statistical parameters (population mean, population standard deviation, and power of the errors) [15, 18] and two main particular cases. First particular case is Gauss distribution [15] often observed on arrays of biochemical data [19–21] while the second particular case is Laplace distribution (with mean of zero and variance *σ* ^2^) [22, 23] commonly seen on astrophysical data [24, 25].

The problem of estimating the parameters of the SLR (1) for the first particular case (Gauss distribution) considers *q* = 2 residuals (where *q* is the power of the errors related with experimental errors). The coefficients of regression for this particular case are obtained by solving the system of linear equations under the assumption that ∑*S*_*i*_^2^ = min [26] (∑*S*_*i*_^2^ = ∑(*Y*_*i*_ − *a* · *X*_*i*_ − *b*)^2^, where *a* and *b* are unknown parameters).

The second particular case is *q* = 1 when residuals follow the Laplace distribution. In view of the fact that ∑ | *S*_*i*_ | = ∑ | *Y*_*i*_ − *a* · *X*_*i*_ − *b*| “is not differentiable everywhere” [27], the solution in more difficult to be obtained for this particular case.

One question can be asked: “what is the proper value of *q* that should be used in the simple linear regression analysis (1)?” A previous study showed that, for different sets of biological active compounds, the distribution of the dependent variable (*Y*) can be approximated by Gauss distribution (*q* = 2) just in a relatively small number of cases when the whole Gauss-Laplace family is investigated [28]. Based on this result, the aim of the present study was to formulate the problem of solving the simple linear regression equation (1) without making any assumptions about the power of the errors (*q*).

## 2. Materials and Methods

### 2.1. Mathematical Approach

The problem of regression (1) is transformed into a problem of estimation if the residuals (*S*_*i*_ = *Y*_*i*_ − *a* · *X*_*i*_ − *b*) are introduced in (2) with a slight modification: in the quantity (*Y*_*i*_ − *a* · *X*_*i*_ − *b*) − *μ* the constants *b* and *μ* are equivalent and just one (*b*) will be further used. Gauss-Laplace distribution is symmetrical and the observed mean is an unbiased estimator of the population mean (*μ* = *b*). This could be expressed in terms of (1) as presented in

(3) $$\begin{array}{c} M\left(Y\right)\sim M\left(\hat{Y}\right)=a\cdot M\left(X\right)+b, \end{array}$$

where *b* is the population mean of the Gauss-Laplace quantity *Y* − *a* · *X* (2), *Y* is observed/measured dependent variable, $\hat{Y}$ is dependent variable estimated by the regression model, *X* is independent/predictor variable, and *M* is mean operator. For certain arrays of paired observations (*X*, *Y*), the problem of regression expressed in (1) is transformed to a problem of estimating the parameters of the bidimensional Gauss-Laplace distribution as presented in

(4) $$\begin{array}{c} \text{GL}\left(x,y;\sigma ,q,a,b\right)=\frac{q}{2\sigma }\frac{\Gamma^{1/2}\left(3/q\right)}{\Gamma^{3/2}\left(1/q\right)}\exp\left(-\frac{{\left|\left(\left(y-a\cdot x\right)-b\right)/\sigma \right|}^{q}}{{\left(\Gamma \left(1/q\right)/\Gamma \left(3/q\right)\right)}^{q/2}}\right). \end{array}$$

An efficient instrument to solve (4) is maximum likelihood estimation (MLE), method proposed by Fisher [16, 17]. The main assumption of the MLE is that the (*X*, *Y*) array has been observed due to its higher chance to be observed (simultaneously and independent). This could be translated as GL(*X*_*i*_, *Y*_*i*_; *σ*, *q*, *a*, *b*) = max, and thus log⁡(ΠGL(*X*_*i*_, *Y*_*i*_; *σ*, *q*, *a*, *b*)) = max, which lead to the expression in

(5) $$\begin{array}{c} \sum \log\left(\text{GL}\left(X_{i},Y_{i};\sigma ,q,a,b\right)\right)=max. \end{array}$$

By including (4) in (5) and using the natural logarithm, the problem presented in (1) became a problem of optimization:

(6) $$\begin{array}{c} \overset{N}{\underset{i=1}{\sum }}\ln\left(\text{GL}\left(X_{i},Y_{i};\sigma ,q,a,b\right)\right)=N\cdot \ln\left(\frac{q}{2\sigma }\frac{\Gamma^{1/2}\left(3/q\right)}{\Gamma^{3/2}\left(1/q\right)}\right)-\frac{\sum_{i=1}^{N}{\left|\left(Y_{i}-a\cdot X_{i}\right)-b\right|}^{q}}{\sigma^{q}{\left(\left(\Gamma \cdot \left(1/q\right)\right)/\left(\Gamma \cdot \left(3/q\right)\right)\right)}^{q/2}}=\text{max.,} \end{array}$$

where *N* is number of (*X*, *Y*) pairs.

The optimization problem presented in (5) could be iteratively solved if the start point is a good initial solution (situated near the optimal solution). In this research, the start point in the optimization was the solution of a particular case of (6) as presented in

(7) $$\begin{array}{c} q=2;\\ a=\frac{\left(M\left(XY\right)-M\left(X\right)\cdot M\left(Y\right)\right)}{D^{2}\left(X\right)}\\ \mu =M\left(Y\right)-a\cdot M\left(X\right)\\ \sigma ={\left(\frac{{D^{2}\left(Y\right)-\left(M\left(XY\right)-M\left(X\right)\cdot M\left(Y\right)\right)}^{2}}{D^{2}\left(X\right)}\right)}^{1/2}, \end{array}$$

where *q* is power of the errors, *μ* is population mean, *σ* is population standard deviation, *M* is average (central tendency operator), and *D* ^2^ is variance (dispersion operator).

### 2.2. Algorithm Implementation

The classical simple linear regression uses least squares method to estimate *a*, *μ*, and *σ* coefficients in (7) using the fixed values of 2 for the power of the errors (*q* = 2). In our approach, starting with the optimal solutions for *a*, *μ*, and *σ* coefficients obtained by (7), the optimal solution of (6) was iteratively obtained by making small changes to the values of the coefficients and selecting the coefficients that make the MLE value higher. The implemented weights of changes were more or less arbitrary, and the selected ones are a compromise of convergence speed in the convergence space.

**Figure 1:**
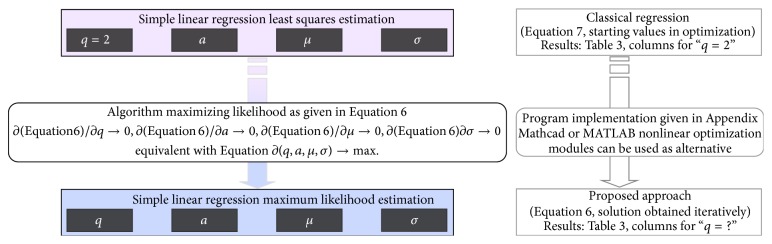
Flowchart of the implemented method. The starting values of the “*a*” (coefficient of the independent variable), “*μ*” (population mean), and “*σ*” (population standard deviation) coefficients are those obtained by least squares estimation method while the imposed value of power of the errors is equal to 2. The algorithm that maximizes likelihood finds optimal solution for “*q*,” “*a*,” “*μ*,” and “*σ*” that satisfy (6).

The flowchart of the proposed approach is presented in Figure 1.

A PHP program was developed to find the optimal solution for (6). As the input data, the implemented program needs a *∗*.txt file with three columns (file named as mol-*X*-*Y*, where* mol* is the identification of the molecule and could be text or number, *X* is the independent variable, and *Y* is dependent variable). The program generates the output file as specified by the user (a *∗*.txt file could be used) that contains for each iteration the data for the following coefficients: *q*, *a*, *μ*, *σ*, and* MLE*.

The source code of the implemented algorithm is free to be used and is presented in the Supplementary Material available online at http://dx.doi.org/10.1155/2015/360752. The full program can be obtained upon request from the authors.

### 2.3. Data Sets

Ten classes of previously investigated compounds were used to assess the proposed method. The class of compounds, the activity/property of interest along with the number of compounds in the dataset and the reference to the paper from where the independent and dependent variables were collected are given in Table 1.

Simple linear regression (SLR) models under the assumption of linear relationship between structural descriptors and activity/property of chemical compounds were identified using the values of descriptors previously published in the literature (see reference in Table 1). The characteristics of the models with the highest goodness-of-fit for each class of compounds are presented in Table 2.

## 3. Results and Discussion

The proposed solution for solving the simple linear regression without making any assumptions about the power of the errors has been successfully implemented and reliable solutions were obtained.

The developed algorithm was successfully tested on ten different data sets. The number of iteration needed to find the optimal solution varied from 9 (set10) to 185 (set4b) and seems not related with the number of compounds in the sample when the same class of compounds is investigated (63 iterations (set1a), 51 iterations (set1b), and 86 iterations (set1c)). The number of iterations needed to obtain the optimal solution was equal to 173 for the smallest dataset (set2) and 86 for the dataset with the highest number of compounds (set1c). Accordingly, the maximum number of iterations was almost 21 times more than the minimum number of iterations.

The results of simulation study obtained for the convenient solution (*q* = 2, residual follows the Gaussian distribution) and for solution that satisfies (6) are presented in Table 3. The values of calculated coefficients (*a*, *b*, and *σ*) are provided with three decimals; equal values for *q* = 2 and optimal *q* were obtained as follows: *a*, coefficient in set1b, set3, and set6; *b*, coefficient in set3, set6, set8, and set10; and *σ*, coefficient in the following sets: 1b, 1c, 3, 4a, 5, 6, 8, 9, and 10.

The analysis of the obtained coefficient presented in Table 3 revealed the following.

- In 9 out of 13 cases, at least one coefficient (*a*, *b*, or *σ*) proved equal for convenience; *q* = 2 and *q* is determined to satisfy (6).
- In 6 out of 13 cases, the power of the errors obtained by MLE proved significantly higher than 2. The difference varied from 0.8099 (set4a) to 7.5176 (set1a).
- Just in one case, the difference between powers of the errors proved not statistically different (set3, *P* = 0.0693).
- In 6 out of 13 cases, the difference between power of the errors (SLR-LS and SLR-MLE) proved lower than 1.
- The smallest distance between the powers of the errors (from SLR-LS and SLR-MLE) was of 0.2613 (set10) and was identified as being statistically significant (*P* < 0.0001).
- Two classes of compounds (set3 and set6) proved identical values of *a*, *b*, and *σ* unconcerned with the method used in the regression analysis (SLR-LS and SLR-MLE).
- The *q* obtained by SLR-MLE proved significantly different by convenient value (*q* = 2) with one exception represented by set3.

The most probable distribution of the power of the error obtained by MLE is Fatigue Life or Birnbaum-Saunders distribution [44] (Kolmogorov-Smirnov statistics = 0.1245, *P* = 0.9728; Anderson-Darling statistics = 0.2753 *P* = 0.9509; *P* value associated with Anderson-Darling statistics was calculated taking into account the values of the statistics and the sample size [45]). The Fatigue Life distribution of the power of the errors is characterized by two parameters represented by continuous shape parameter (*α* = 0.7777) and continuous scale parameter (*β* = 2.0599). The median of the power of the errors is closed to the convenient values of 2, with a mean of 2.68. Nevertheless, the normal distribution of the obtained power of the errors could not be rejected at a significance level of 5% (Kolmogorov-Smirnov statistics = 0.278, *P* = 0.2229; Anderson-Darling statistics = 1.178, *P* = 0.2731).

**Figure 2:**
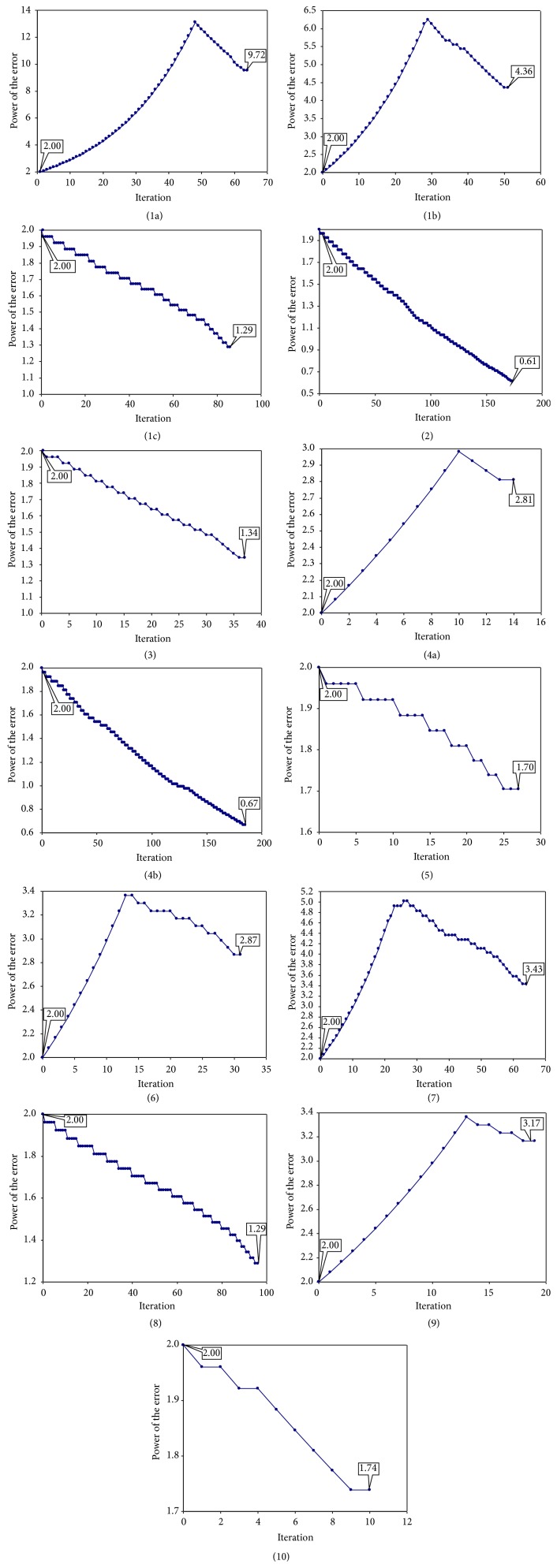
Distribution of power of the errors according to iteration: investigation of phenols set (35 compounds (1a) and 126 compounds (1b), resp.). Distribution of power of the errors according to iteration: phenols (1c), organic compounds (2), alkanes (3), flavonoids (4a and 4b), estrogen receptor (5), pyrrolo-pyrimidine derivatives (6), and substituted aromatic sulfonamides (7). Distribution of power of the errors according to iteration: behavior on carboquinone derivatives (8), dipeptides (9), and mycotoxins compounds (10).

The evolution of value of power of the errors according to iteration was in both directions and, as expected, never achieved negative values (see Figure 2). The analysis of the evolution of the power of the errors as function of iteration revealed that even if identical values of *q* are obtained in the first 29 iterations for the first two related samples (set1a and set1b, Figure 2), the pattern is not representative for the class of the compounds. Thus, the pattern from 1c is significantly different by those observed on subsets of the whole class of compounds (1a and 1b). Opposite behavior is also observed for the other two related samples (set4a and set4b), and the value of *q* increased until a maximum (iteration 10 for set4a) and decreased after this value while the value of *q* decreases in steps for set4b.

Overall, two distinct patterns are observed in Figure 1. In the first pattern, the values of power of the error increase with iteration until a peak and after that the value decreases (sometimes with a decrease in steps (set6, set7, and set9)); see set1a, set1b, set4a, set6, and set9 (Figure 2). In the second pattern, the power of the error decreases in steps with the increase of iteration as for set1c, set2, set3, set4b, set5, set8, and set10 (see Figure 2).

**Figure 3:**
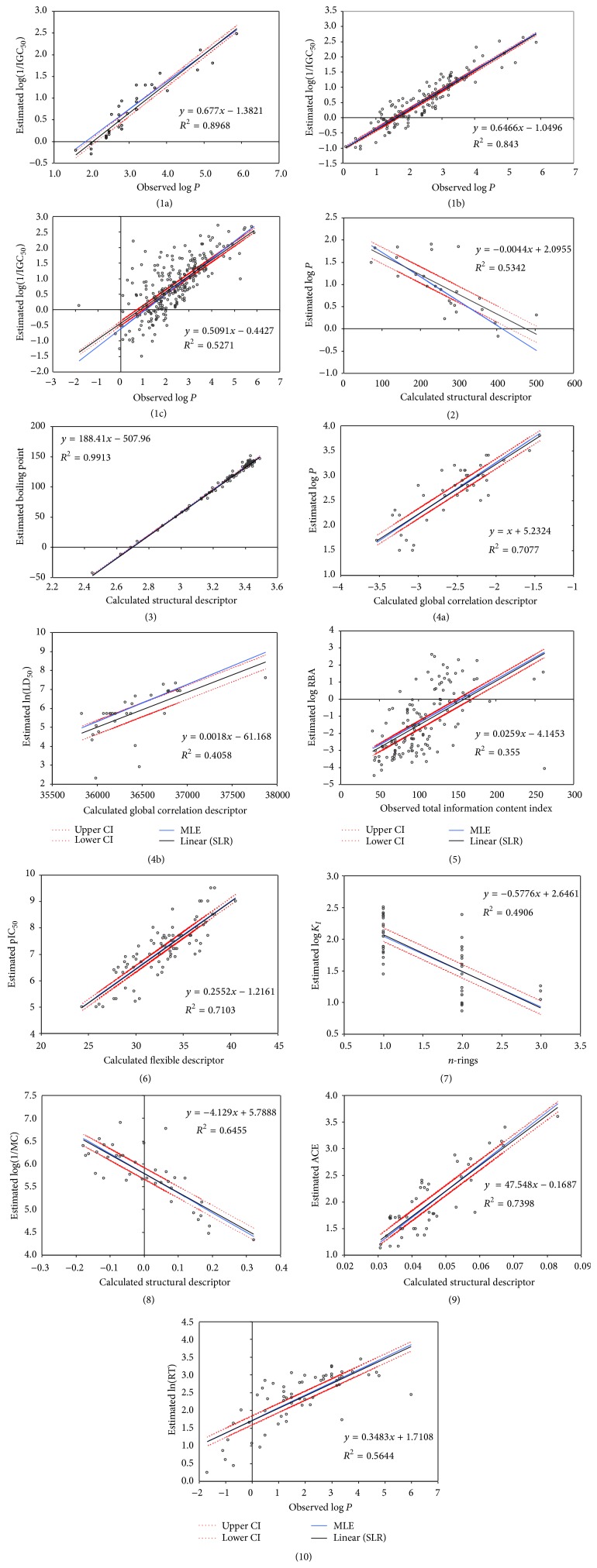
The line of SLR-LS (*q* = 2) and SLR-MLE (*q* determined to satisfy (6)): investigation of phenols set (35 compounds (1a) and 126 compounds (1b), resp.). Phenols (1c), organic compounds (2), alkanes (3), flavonoids (4a and 4b), estrogen receptor (5), pyrrolo-pyrimidine derivatives (6), and substituted aromatic sulfonamides (7). Carboquinone derivatives (8), dipeptides (9), and mycotoxins compounds (10).

The plot of both regression lines (simple linear regression and associated 95% confidence interval and MLE regression) for each investigated data sets is presented in Figure 3.

The analysis of the regression lines presented in Figure 2 revealed that, in one case represented by set7, the assumption of the linearity of log⁡*K*_*I*_ with* n-rings* is breached and, for this dataset, the simple linear regression is not the proper analysis. In 4 out of 13 cases, the SLR-MLE line is partly outside the 95% confidence boundaries of the SLR-LS line (set1a, set1c, set2, and set4b; Figure 3). Accordingly, it could be considered in all these cases that the SLR-MLE model is significantly different by the SLR-LS model. The overlapping of SLR-MLE and SLR-LS line is observed for the set3, without being possible to make a visual distinction between them (Figure 3). For this set, the *q* obtained by SLR-MLE was equal to 1.34 and proved not significantly different by convenient value of 2 (see Table 3). For all other sets, the SLR-MLE line is within the boundaries of 95% confidence intervals of SLR-LS line and thus even if the powers of the errors proved significantly different by the convenient value of 2, these SLR-MLE models could not be considered significantly different by the SLR models.

To conclude, it is certain that the proposed approach of maximizing the probability of observing the event according to the random error fits well the observed data and frequently the power of the errors (*q*) is significantly different by the convenient value (*q* = 2). However, no pattern could be identified between iteration and sample size on the investigated sets of (*X*, *Y*) pairs. It is expected that the recognized behavior of the power of the errors is to be identified on other (*X*, *Y*) pairs, analysis which is currently conducted by our team. The relation presented in (6) thereby defines a new general approach to treat the relationships. Practically, the expression *S*_*i*_ = *Y*_*i*_ − *aX*_*i*_ could be replaced with any expression of dependency (not just linear), such as

- exponential: *S*_*i*_ = *Y*_*i*_ − *a*_1_ · exp⁡(−*X*_*i*_/*a*_2_) for *Y* ~ *a*_0_ + *a*_1_ · exp⁡(−*X*/*a*_2_);
- double exponential: *S*_*i*_ = *Y*_*i*_ − *a*_1_ · exp⁡(−*X*_*i*_/*a*_2_) − *a*_3_ · exp⁡(−*X*_*i*_/*a*_4_) for *Y* ~ *a*_0_ + *a*_1_ · exp⁡(−*X*/*a*_2_) + *a*_3_ · exp⁡(−*X*/*a*_4_);
- power: *S*_*i*_ = *Y*_*i*_ − *a*_1_ · pow(*X*_*i*_, *a*_2_) for *Y* ~ *a*_0_ + *a*_1_ · pow(*X*, *a*_2_);
- inversed: *S*_*i*_ = *Y*_*i*_ − *a*_1_/(*X*_*i*_ − *a*_2_) for *Y* ~ *a*_0_ + *a*_1_/(*X* − *a*_2_).

The relation presented in (6) may be also extended to the multiple linear regression (*Y* ~ *a*_0_ + ∑_*j*>0_ *a*_*j*_ *X*_*j*_) when the expression *S*_*i*_ = *Y*_*i*_ − *aX*_*i*_ becomes *S*_*i*_ = *Y*_*i*_ − ∑_*j*>0_ *a*_*j*_ *X*_*j*,*i*_. If in the case of multiple linear regressions the classical method (minimizing the squared error) maximizes the correlation coefficient, the proposed approach (6) maximizes the probability of observing the event according to the random error. In view of that, (6) has a significant advantage compared to the classical approach. The classical approach that maximizes the correlation coefficient is exposed to type I errors; a model of regression could be accepted even if the model does not exist. On the contrary, the proposed approach that maximizes just the chance of observation (the approach has just one hypothesis: the error between the observation (*Y*) and the model $\left(\hat{Y}\right)$ must be random and its value does not depend on the size of the observed value) is not affected by a type I error. In the case of simple linear regression, application of (6) did not change the correlation coefficient between *Y* and $\hat{Y}$ but offers a solution in regard to estimated valued of *Y* and of the unknown coefficients (estimators of the population coefficients) that enter the relation between *X* and *Y*. The relation proposed in this paper (6) introduced an additional parameter in the estimation, namely, the power of the errors of Gauss-Laplace distribution (*q*) (this led to decrease by one unit of the degrees of freedom in the analysis of variance in the regression model).

The MLE approach is frequently used in estimation of unknown parameters and it is known to be sensitive to outliers (±influential compounds) in the data [46–48]. No outliers have been identified in the dependent variable on set2 and set3 [42, 46, 47]. Therefore, on these two sets of compounds, it is a certainty that the proposed approach was not affected by the presence of outliers in the data. Evaluation of how the values in the investigated sets could lead to identification of outliers (±influential compounds [4, 31, 49]) was beyond the aim of the present study. The proposed approach proved its usefulness in estimation of SLR parameters and is now under evaluation by our team on different types of classes of compounds and relations to assess its behavior and robustness.

## 4. Conclusions

The proposed approach proved feasible for estimating the parameters of the simple linear regression, in the absence of the assumption that the errors are normally distributed, assumption replaced by a more general one that the errors are Gauss-Laplace distributed. The obtained results demonstrated that in 12 out of 13 investigated cases the power of the error is significantly different by the convenient values of two. However, the plot of SLR-MLE and SLR-LS lines showed that, just in 3 out of 12 cases, the models are significantly different. The proposed approach can be further extended from simple linear regressions to multiple linear regressions.

## Supplementary Material

The classical simple linear regression (SLR) uses least squares method to estimate a, µ and s coefficients (see Eq7) using the value of the power of the errors equal to 2. The supplementary material contains lines of the program implemented in PHP to find the solutions of Eq6 (maximum likelihood estimation - MLE) starting with values of coefficients identified by Eq7. The program makes small changes to the values of the coefficients and selects the coefficients that maximize the MLE value.

## Figures and Tables

**Table 1:** Characteristics of the investigated classes of compounds.

Set	*n*	Class	Activity/property, expressed as	Reference
1a	35	Phenols	Toxicity on *Tetrahymena pyriformis*, log⁡(1/IGC_50_)	[29–31]
1b	126			
1c	250			

2	24	Organic compounds	Solubility, log⁡*P*	[32, 33]

3	73	Alkanes	Boiling point, BP	[34]

4a	40	Flavonoids	Solubility, log⁡*P*	[35]
4b	30		Lethal Dose 50%, ln⁡(LD_50_)	

5	132	Estrogen receptor (ER)	Binding affinities, log⁡(RBA)	[36]

6	80	Pyrrolo-pyrimidine derivatives	c-Src tyrosine kinase inhibitory activity, pIC_50_ = −log_10_⁡(IC_50_)	[37]

7	47	Substituted aromatic sulfonamides	Inhibition activity on carbonic anhydrase II, log⁡*K*_*I*_	[38]

8	37	Carboquinone derivatives	Molar concentration, log⁡(1/MC)	[39]

9	47	Dipeptides	ACE (angiotensin converting enzyme) inhibitory activity, ACE	[40]

10	60	Mycotoxins compounds	Retention time, ln⁡(RT)	[41]

**Table 2:** Characteristics of the SLR-LS models used in the optimization study.

Set	SLR model	*R* ^2^	*s*	*F*	*n*
1a	log⁡(1/IGC_50_) = +0.677 · log⁡*P* − 1.38	0.90	0.22	287	35
1b	log⁡(1/IGC_50_) = +0.647 · log⁡*P* − 1.05	0.84	0.30	666	126
1c	log⁡(1/IGC_50_) = −0.443 · log⁡*P* + 0.509	0.53	0.57	276	250
2	log⁡*P* = −0.004 · ISDRTHg^*∗*^ + 2.09	0.53	0.43	25	24
3	BP = +188.40 · lbMdsHg^*∗*^ − 507.95	0.99	3.81	8050	73
4a	log⁡*P* = +0.99998 · SD + 5.232	0.71	0.32	92	40
4b	ln⁡(LD_50_) = +0.0018 · SD − 61.168	0.41	0.98	19	30
5	log⁡RBA = +0.026 · TIC1 − 4.145	0.36	1.44	72	132
6	pIC_50_ = +0.255 · DCW − 1.216	0.71	0.57	191	80
7	log⁡*K*_*I*_ = −0.578 · *N*-rings + 2.646	0.49	0.37	43	47
8	log⁡(1/MC) = −4.129 · TEuIFFDL^*∗*^ + 5.789	0.65	0.38	64	37
9	ACE = 47.5480 · IHMdpMg^*∗*^ − 0.1687	0.74	0.33	128	47
10	ln⁡(RT) = 0.348 · log⁡*P* + 1.711	0.56	0.50	75	60

SLR = simple linear regression.

log⁡(1/IGC_50_) = concentrations (expressed as mM) producing a 50% growth inhibition on *T. pyriformis*.

^*∗*^MDF descriptors [33, 39, 40, 42].

SD = global correlation descriptor [35]; TIC1 = total information content index (neighborhood symmetry of 1-order).

DCW = flexible (activity dependent) descriptor.

std_dim3 = the square root of the third largest eigenvalue of the covariance matrix of the atomic coordinates [43].

*R* ^2^ = determination coefficient; *s* = standard error of the estimate.

*F* = Fisher's statistic of the regression model; *n* = sample size.

**Table 3:** Optimization results: *q* = 2 versus *q* determined to satisfy (6).

set	*n*	*q* = 2			*q* = ?				*P* value (*H*_*o*_: *q* = 2)
		*a*	*b* = *μ*	*σ*	*q*	*a*	*b* = *μ*	*σ*	
1a	35	0.678	−1.386	0.218	9.52	0.638	−1.181	0.222	4.20 · 10^−54^
1b	126	0.647	−1.050	0.298	4.36	0.647	−1.029	0.298	3.07 · 10^−115^
1c	250	0.509	−0.443	0.596	1.29	0.563	−0.623	0.569	2.42 · 10^−53^
2	24	−0.004	2.095	0.414	0.61	−0.005	2.270	0.516	1.76 · 10^−12^
3	73	188.408	−507.959	3.762	1.34	188.408	−507.959	3.762	6.93 · 10^−2^
4a	40	1.000	5.232	0.308	2.81	1.041	5.338	0.308	1.30 · 10^−19^
4b	30	0.002	−61.168	0.945	0.67	0.002	−64.950	0.964	1.16 · 10^−8^
5	132	0.024	−3.812	1.374	1.70	0.026	−3.967	1.374	7.33 · 10^−3^
6	80	0.255	−1.216	0.558	2.87	0.255	−1.216	0.558	3.39 · 10^−23^
7	47	−0.578	2.646	0.360	3.43	−0.555	2.594	0.353	1.06 · 10^−30^
8	37	−4.129	5.789	0.372	1.29	−4.297	5.789	0.372	4.75 · 10^−14^
9	47	47.561	−0.169	0.319	3.17	49.502	−0.279	0.319	9.01 · 10^−29^
10	60	0.348	1.711	0.492	1.74	0.355	1.711	0.492	6.09 · 10^−5^

*q* = power of the errors; *a*, *b* = coefficients in the simple linear model.

*μ* = population mean; *σ* = population standard deviation.
