# Supplementary material for: Inside of the Linear Relation between Dependent and Independent Variables
Source: Comput Math Methods Med. 2015 May 25;2015:360752. doi: 10.1155/2015/360752 (PMC4458545; doi:10.1155/2015/360752)
Supplement: Supplementary file 1 — The classical simple linear regression (SLR) uses least squares method to estimate a, µ and s coefficients (see Eq7) using the value of the power of the errors equal to 2. The supplementary material contains lines of the program implemented in PHP to find the solutions of Eq6 (maximum likelihood estimation - MLE) starting with values of coefficients identified by Eq7. The program makes small changes to the values of the coefficients and selects the coefficients that maximize the MLE value. [file 360752.f1.pdf]

Supplementary Information for "Inside of the Linear Relation between Dependent and Independent Variables"

Lorentz JÄNTSCHI[1,2,3], Lavinia L. PRUTEANU[2], Alina C. COZMA[3,4], Sorana D. BOLBOACĂ[4,*]

[1] Technical University of Cluj-Napoca, Department of Physics and Chemistry, Muncii Bvd. 103-105, 400641 Cluj-Napoca, Romania. E-mail: lorentz.jantschi@gmail.com

[2] Babeş-Bolyai University, Institute for Doctoral Studies, Kogălniceanu Street no. 1,400084 Cluj-Napoca, Romania. E-mail: lavinia@j.academicdirect.org

[3] University of Oradea, Department of Chemistry, Universităţii Street no. 1, 410087 Oradea, Romania. E-mail: acozma@uoradea.ro

[4] Iuliu Haţieganu University of Medicine and Pharmacy, Department of Medical Informatics and Biostatistics, Louis Pasteur Street no. 6, 400349 Cluj-Napoca, Romania. E-mail: sbolboaca@umfcluj.ro

An object (ERegression) was created to solve the problem:

```
class ERegression{
    function DCopy(&x,&y); //initialize with data
    function DStat(); //do the analysis
    function m01(&z); //compute the average of the array
    function m02(&z,&w); //compute the average of the arrays product
    function GLMLE(&it); //calculate MLE of GL for given parameters
    function Steps(); //iterates the convergence to the optimal parameters
    function ERegression(&u,&v){DCopy(u,v); DStat();} //constructor of the object
}
function ERegression.m01(&z){s=0.0;for(i=0;i<m;i++)s+=z[i];s/=m; return(s);}
function ERegression.m02(&z,&w){s=0.0;for(i=0;i<m;i++)s+=z[i]*w[i];s/=m; return(s);}
function ERegression.DCopy(&u,&v){y=&u;x=&v;m=count(y);}
function ERegression.DStat(){
        my1=m01(y);my2=m02(y,y);dy2=my2-pow(my1,2);
        mx1=m01(x);mx2=m02(x,x);dx2=mx2-pow(mx1,2);
        mxy=m02(x,y);cxy=mxy-mx1*my1;
```

```
            guess=array(
                    "p" => 2,
                    "a" => cxy/dx2,
                    "m" => my1-guess["a"]*mx1,
                    "s" => pow(dy2-pow(cxy,2)/dx2,0.5),
                    "MLE" => 0
            );
            guess["MLE"]=GLMLE(guess);
            stepx=(stepn-stepn%2)/2;
            stepv=array();for(i=0;i<stepn;i++)stepv[i]=exp((i-stepx)/50.0);
}
function ERegression.GLMLE(&it){
            g1=gamma1p(it["p"]);
            g3=gamma3p(it["p"]);
            t1=m*log(it["p"]*pow(g3,0.5)/pow(g1,1.5)/it["s"]/2.0);
            t2=pow(g1/g3,it["p"]/2.0)*pow(it["s"],it["p"]);
            t3=0.0;
            for(i=0;i<m;i++){t3+=pow(abs(y[i]-it["a"]*x[i]-it["m"]),it["p"]);}
            return(t1-t3/t2);
}
```

The optimal solution of Eq(6) is iteratively obtained from the optimal solution Eq(7) by making small changes to the actual values of the coefficients and selecting the new ones that makes the MLE value greater. The weights of changes are more or less arbitrary, and the selected ones are a compromise of convergence speed in the convergence space.

```
function ERegression.Steps(){
      itera=array();bestf=guess;
      for(;;){
            for(i1=0;i1<stepn;i1++){ //for p
                    itera["p"]=guess["p"]*stepv[i1];
                    for(i2=0;i2<stepn;i2++){ //for a
                            itera["a"]=guess["a"]*stepv[i2];
                                    for(i3=0;i3<stepn;i3++){//for m
```

```
                        itera["m"]=guess["m"]*stepv[i3];
                        for(i4=0;i4<stepn;i4++){//for s
                            itera["s"]=guess["s"]*stepv[i4];
                            itera["MLE"]=GLMLE(itera);
                            if(itera["MLE"]>guess["MLE"])bestf=itera;
                        }
                    }
                }
            }
            itera=bestf;
            if(abs(itera["MLE"]-guess["MLE"])<stope)break;
            guess=itera;
        }
    }
}
```

The values of dependent (*Y*) and independent (*X*) variables are read from a given **.txt* file and the array of *X* and *Y* is returned through the function named **`get_data`**:

```
function get_data($text){
    $a=explode("\r\n",file_get_contents($text)); //get the data from the text file
    array_shift($a); //chop the headers
    $x=array(); $y=array(); //initialize with empty the arrays of observed values
    for($i=0; $i<count($a); $i++){ //for each line containing data
        $b=explode("\t",$a[$i]); //split the line into the pair of x and y values
        $x[]=$b[1]; $y[]=$b[2]; //collect x and y values
    }
    return(array($x,$y)); //return arrays of values
}
```

The main part of the program calls the ERegression object to find the solution to a given dataset:

```
$xy=get_data("some_file_name.txt"); //return the data as an array of two arrays
$reg_xy = new ERegression($xy[0],$xy[1]); //instantiate the ERegression object
```

```
$reg_xy->Steps(); //iterate the optimal solution
```

The source code of the implemented algorithm is freely to be used.
